# Mesh Suture and Mesh Strips to Prevent Incisional Hernia Following Abdominal Wall Closure or Ventral Hernia Repair: Systematic Review

**DOI:** 10.3389/jaws.2025.14573

**Published:** 2025-05-14

**Authors:** Lawrence Nip, Sarah Zhao, Rhys Thomas, Alastair C. J. Windsor, Sue Mallett, Steve Halligan, Samuel G. Parker

**Affiliations:** ^1^ The Abdominal Wall Unit, Croydon University Hospital, London, United Kingdom; ^2^ Centre for Medical Imaging, Division of Medicine, University College London, London, United Kingdom; ^3^ The Princess Grace Hospital, HCA Healthcare, London, United Kingdom

**Keywords:** ventral hernia repair, laparotomy closure, Duramesh, mesh suture, incisional hernia

## Abstract

**Background:**

Mesh suture, or Duramesh™, has recently gained attention because of potential advantages over conventional techniques for abdominal wall closure. However, the evidence base for any advantage has not been assessed formally. Via systematic review we evaluated clinical outcomes for mesh suture and its precursor, mesh strip, in clinical trials of abdominal wall closure or ventral hernia repair.

**Methods:**

A systematic search of MEDLINE, CENTRAL, Embase, Cochrane, WHO International Clinical Trials Registry, and ClinicalTrials.gov was conducted to identify studies using mesh suture and/or mesh strip. Primary outcome was incisional hernia occurrence after primary closure or recurrence following ventral hernia repair, summarised with median percentage rates. Secondary outcomes included surgical site occurrences and reoperations. Risk of bias was assessed using adapted forms of ROBINS-I and Cochrane RoB2 tools.

**Results:**

Five single-arm case series and one interim report from a randomised controlled trial were eligible for inclusion, reporting 585 patients. Median follow-up was 11.9 months (range 2.7–35.3 months). Median incisional hernia occurrence was 3.4% (range 0%–50%). Median surgical site occurrence was 17.4% (range 0%–50%) and surgical site infection 5.4% (range 0%–19%). Overall, 6.0% patients (33 of 553) returned to theatre to manage complications. Overall risk of bias for included studies was critical.

**Conclusion:**

This systematic review highlights a need for high-quality randomised controlled trials with long-term follow-up to evaluate the clinical benefits of Duramesh™ for abdominal wall closure and ventral hernia repair. Better evidence is required to determine its safety and clinical efficacy.

## Introduction

Incisional hernias are an increasingly common complication encountered by surgeons and their patients, as survival rates from major abdominal surgery continue to improve. The incidence of incisional hernia after midline laparotomy is estimated to be 9%–20% after 1 year [1], resulting in approximately 8000 UK repairs annually [2]. While patient factors such as obesity, smoking and diabetes certainly contribute, excessive suture tension during the critical wound healing period causes local ischaemia at the suture-tissue interface and may initiate incisional hernia [3]. Subsequent suture “cheese-wiring” through fascia creates small linear defects that enlarge over time with repeated abdominal wall straining. The clinical and economic implications of incisional hernia have precipitated preventative research, including Jenkins rule [4], small-bite closure [5], and prophylactic mesh implantation [6] which are discussed in recent high-profile international guidelines [7, 8]. However, many surgeons continue to adopt suture closure over prophylactic planar mesh since this prolongs surgery and risks infection in a contaminated field.

**FIGURE 1:**
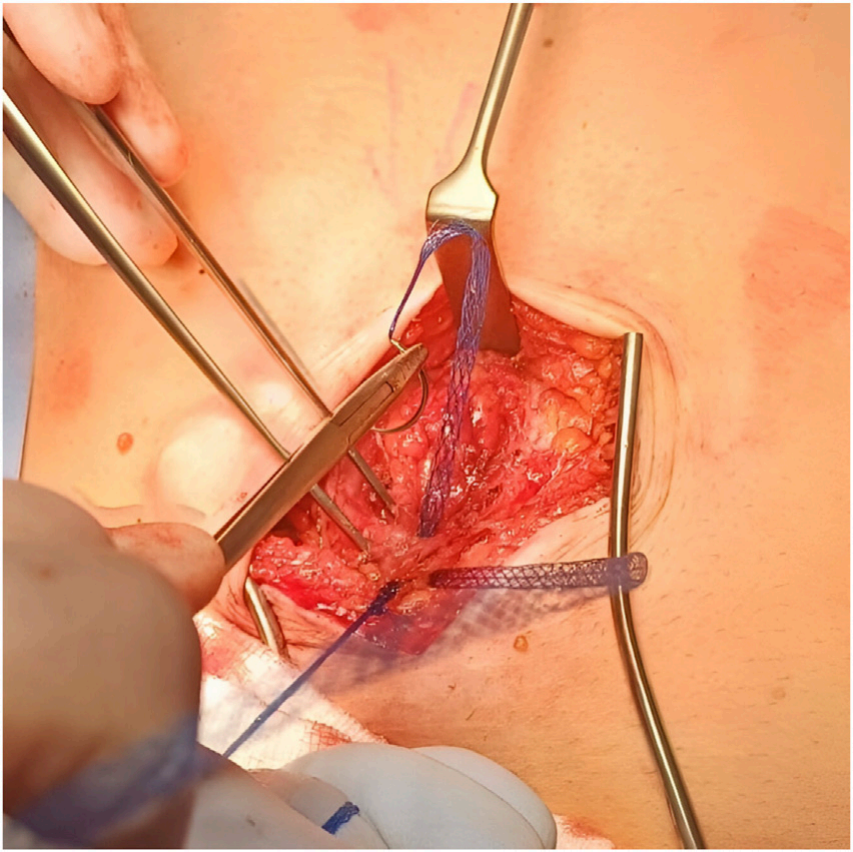
Intraoperative photo showing Duramesh™ in use.

Dumanian, a general surgeon with specialty training in plastic surgery, recently created a novel mesh suture (Duramesh™) intended to solve the biomechanical problem of suture pull-through, and was approved for humans, May 2021 and September 2022 in the EU/UK and US respectively. Duramesh™ (Figure 1) is a cylindrical latticework of polypropylene filaments attached to the swage-end of a standard curved needle which, upon tying, flattens out, emulating a ribbon of planar mesh, thereby distributing tension at the suture-tissue interface and allowing fibrovascular ingrowth around individual filaments to strengthen repair [9]. Preclinical studies have shown that Duramesh™ requires greater ultimate tensile strength and maximal force to cause suture pull-through, compared to 0-polyprolyene suture [9, 10]. Proponents of Duramesh™ report that it combines the simplicity and speed of suture closure while simultaneously reducing hernia occurrence and surgical site infection (SSI) typically seen with prophylactic mesh [6].

Mesh strips were the first iteration of mesh suture, consisting of planar mesh cut into 2 cm strips and tied like conventional suture to achieve tissue apposition. Animal models found improved resistance to pull-through and incisional hernia formation [11, 12], and several subsequent studies demonstrated safety in human primary umbilical hernia repair [13], contaminated incisional hernia repair [14], and repair of large abdominal wall defects [15]. Recognising growing enthusiasm amongst the abdominal wall community, we performed a scoping literature search that suggested the current evidence base for mesh suture and strips typically cites case-series, reporting relatively few patients; we decided to investigate this further via systematic review. At the time of writing, we believe this is the first systematic review of mesh suture and mesh strips for abdominal wall closure and ventral hernia repair.

## Methods

### Objectives

This systematic review aimed to investigate postoperative outcomes associated with mesh suture and mesh strips compared to conventional suture, for abdominal wall closure. The primary outcome of interest was incisional hernia occurrence after primary closure or recurrence following ventral hernia repair. Secondary outcomes included surgical site occurrence and surgical site infection as defined by the Centers for Disease Control and Prevention (CDC) and Ventral Hernia Working Group [16, 17]. Other clinically relevant outcomes including mortality, return to theatre, length of hospital stay, and patient reported outcome measures (PROMs) were also extracted.

### Registration and Reporting

This systematic review was performed and reported according to PRISMA guidelines [18]. Ethical approval is not required by our centre for systematic reviews of available medical literature. Our protocol was registered with the International Prospective Register of Systematic Reviews (PROSPERO) with registration number: CRD42024529173. The original review protocol is available on request from the corresponding author, but an abbreviated version can be found by searching the above registration number on PROSPERO. No amendments were made to the protocol after it had been written and endorsed by all co-authors. Our protocol was finalised prior to literature search and data collection.

### Inclusion and Exclusion Criteria

We studied adults 18 years and over, undergoing ventral abdominal wall closure for any indication in elective or emergency settings. While we anticipated most studies would report outcomes following midline closure, we did not restrict to this and included off-midline and port-site closures. Primary, incisional and recurrent ventral hernias, including repair in contaminated fields, were eligible as long as repair did not involve planar mesh or prophylactic planar mesh following primary closure.

Exclusion criteria included parastomal hernia repair, groin hernia repair, rectus diastasis repair, limb tendon repair, and repair involving bone. Additionally, we excluded articles where the full text was unavailable or not in English, descriptions of surgical technique (“how to” articles), conference abstracts, expert opinion, editorial, and case reports of 5 patients or less.

### Search Strategy and String

An electronic database search of MEDLINE, CENTRAL, EMBASE and Cochrane from 1st Jan 2000 to 28th Feb 2024 was conducted by the first author. The WHO International Clinical Trials Registry and ClincialTrials.gov databases were searched for any ongoing and/or unpublished interventional studies. An electronic database search of the grey literature was not performed. Where identified, corresponding authors were contacted. Industry representatives and 2 hernia opinion leaders were also contacted to source further eligible data not identified by electronic databases. Industry representatives were selected from known suppliers of Duramesh™ in the UK, and hernia opinion leaders were selected if they gave a keynote talk about Duramesh™ at an international hernia conference. Reference lists of all included articles were manually cross searched to source any additional studies.

Our search string to identify relevant articles included the terms “mesh suture” and “abdominal wall closure” combined by the Boolean operator “AND”. Synonyms and related terms such as “laparotomy,” “ventral hernia repair” and “incisional hernia repair” were encompassed by the Boolean operator “OR.” Complete search strings for each database are provided in appendix 1 (Supplementary Material).

### Article Screening

All eligible citations were uploaded onto a reference manager (Mendeley for Mac, Version 2.110.2, Elsevier, Amsterdam, Netherlands) with duplicates removed. Two authors working independently screened titles and abstracts and excluded any articles clearly unsuitable for this review. Following this, full texts of remaining articles were retrieved and scrutinised for inclusion according to eligibility criteria. Any discrepancies between the authors were arbitrated by a senior member of the research team.

### Data Extraction

For each study, data were extracted and populated onto a spreadsheet designed specifically for the study (Microsoft Excel for Mac, Version 16.66.1, Microsoft Corporation, Washington, United States). Information extracted included: study demographics (author, year, country, journal, study type, dates, sample size, study arms, arm sizes, follow-up), patient demographics (male to female ratio, mean age, mean BMI, diabetes, smoking status), and primary and secondary outcomes as listed above. Imputation of missing data was not performed, and missing values were described as “not reported” if still unavailable after contacting the corresponding authors.

### Statistical Analysis

In the absence of 3 or more homogenous comparative studies for meta-analysis, descriptive tables were created to aid narrative review. Primary and secondary outcomes were summarised with median and range due to the heterogenous dataset. Results were reported in prevalence Forest plots with 95% confidence intervals.

### Risk of Bias

We acknowledged from a scoping literature search that the current evidence base may be restricted to single-arm case series. This was performed by the first author on 20th Jan 2024 to ascertain if there was enough evidence to progress to formal systematic review. Although case series are inherently biased, they may represent the best available evidence, especially for emerging medical technologies [19]. Whilst designing our protocol, we felt it was imperative that hernia surgeons are made aware of the rigorous requirements of performing high-quality trials. Duramesh™ and the concept of mesh suture is gaining traction in clinical practice but is ultimately still a novel product. Therefore, available studies must be critically appraised against the standards required for robust interventional trial methodology. Consequently, risk of bias was assessed using an adapted form of the Risk of Bias in Non-Randomised Studies of Interventions (ROBINS-I) tool for any identified observational studies including case series [20], and the Cochrane Risk of Bias 2 tool for randomised controlled trials [21]. Target trial methodology was employed with the use of hypothetical control arms for single arm studies. A detailed description of our adapted risk of bias assessment is provided in the Supplementary Material.

## Results

**FIGURE 2:**
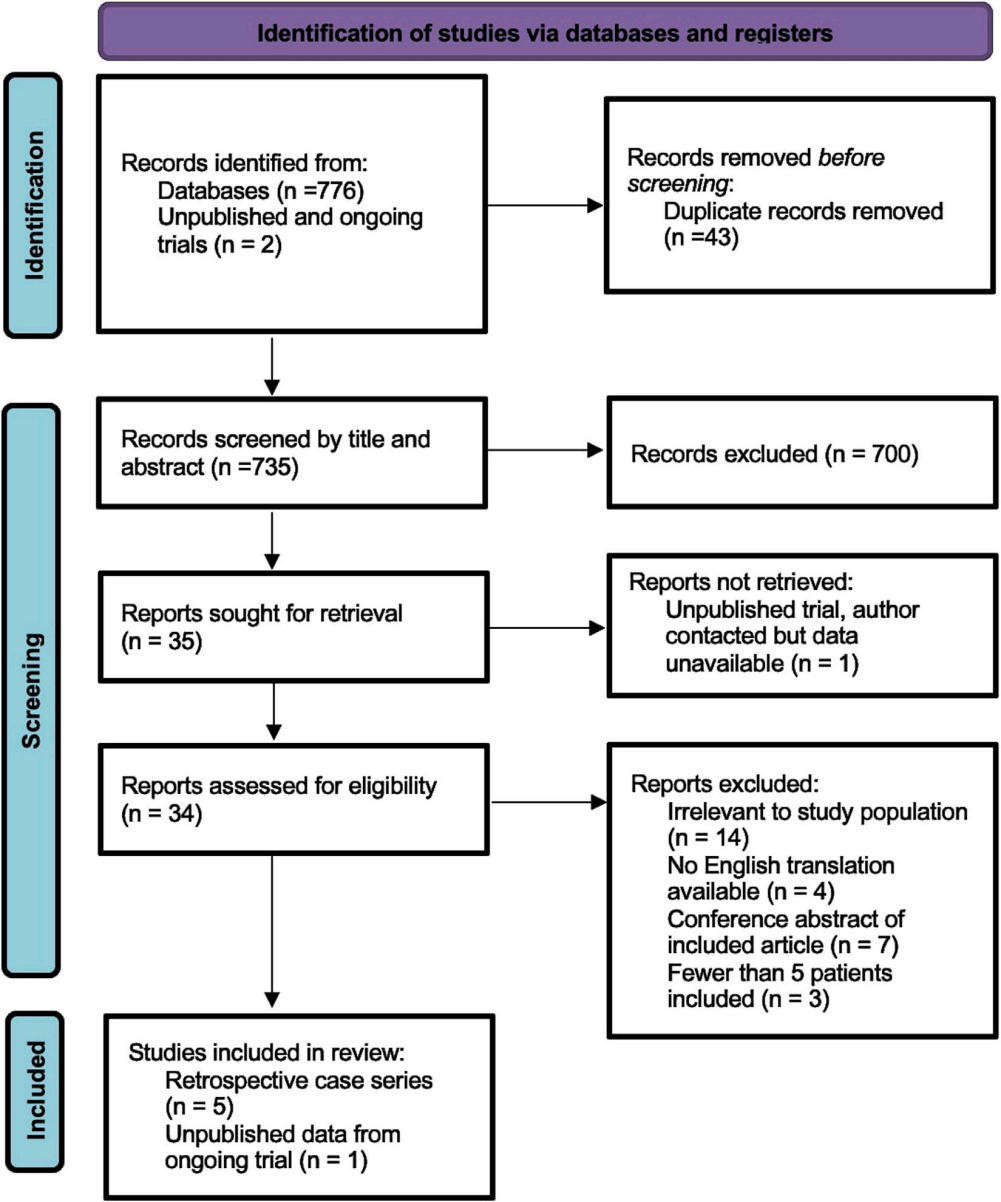
PRISMA flow diagram showing the number of records identified and excluded at each stage.

The search identified 778 references. After removing 43 duplicates, the title and abstract of 735 studies were screened and 701 excluded. 34 manuscripts were examined in full of which six were suitable for inclusion [13–15, 22–24]. Five were retrospective case series (four single surgeon series [13–15, 22], one pooled database of multiple surgeons [24]), and one was an interim report from an ongoing randomised controlled trial [22]. Our PRISMA flow diagram is shown in Figure 2. In total, there were 585 patients reported of which 553 (95%) underwent mesh suture or mesh strip repair, and 32 patients underwent conventional suture repair during open surgery. There were no studies investigating laparoscopic or robotic trocar site closure. Meta-analysis was therefore not conducted due to heterogeneity of participants and low number of participants undergoing conventional suture repair.

### Risk of Bias Assessment

**FIGURE 3:**
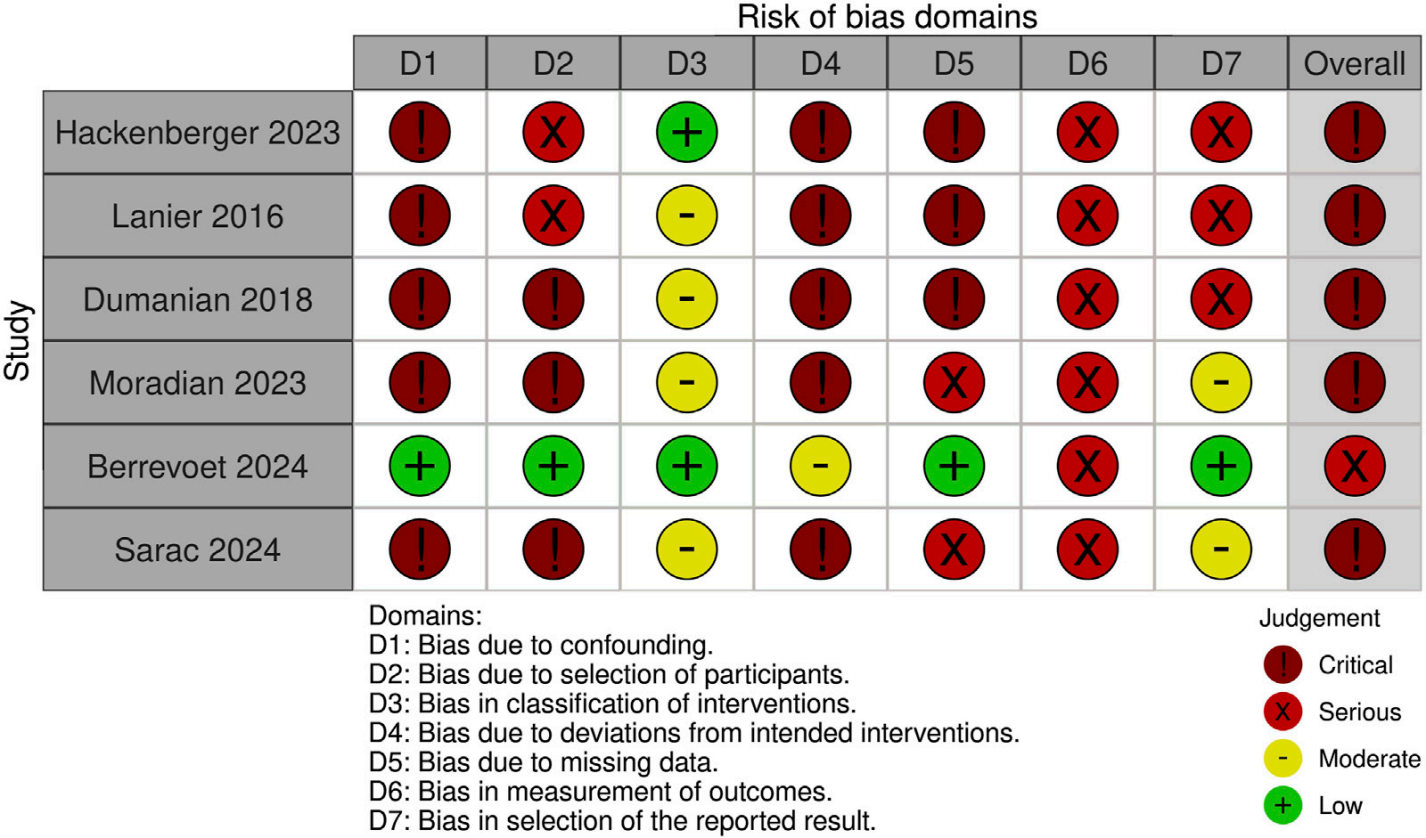
Authors’ judgement for each risk of bias domain using an adapted form of the ROBINS-I tool.

**FIGURE 4:**
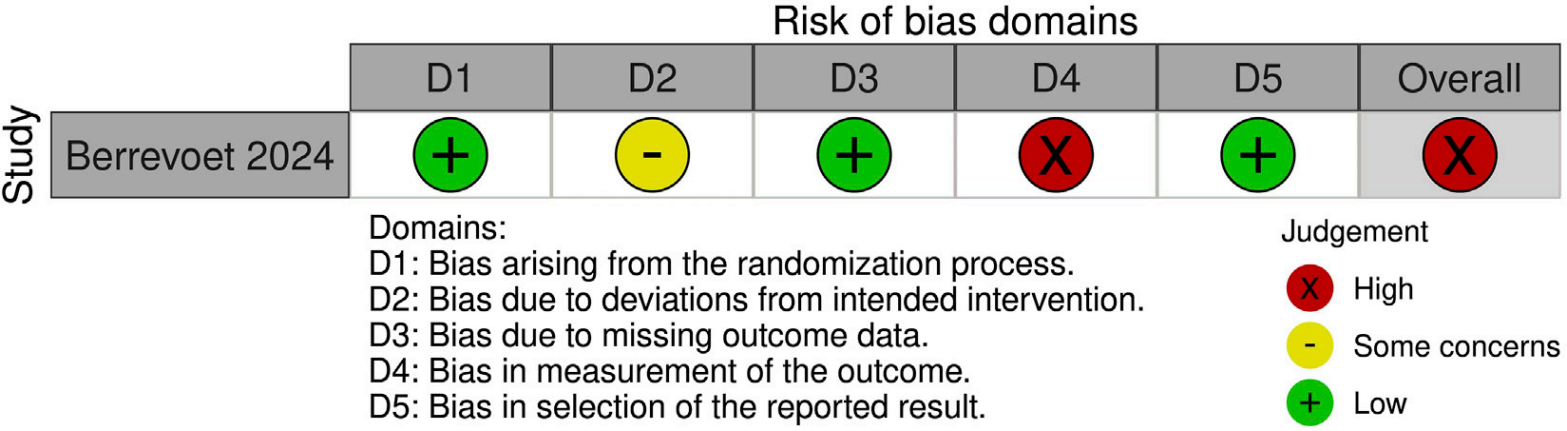
Risk of bias assessment of included randomised controlled trials using the Cochrane Risk of Bias 2 tool.

**FIGURE 5:**
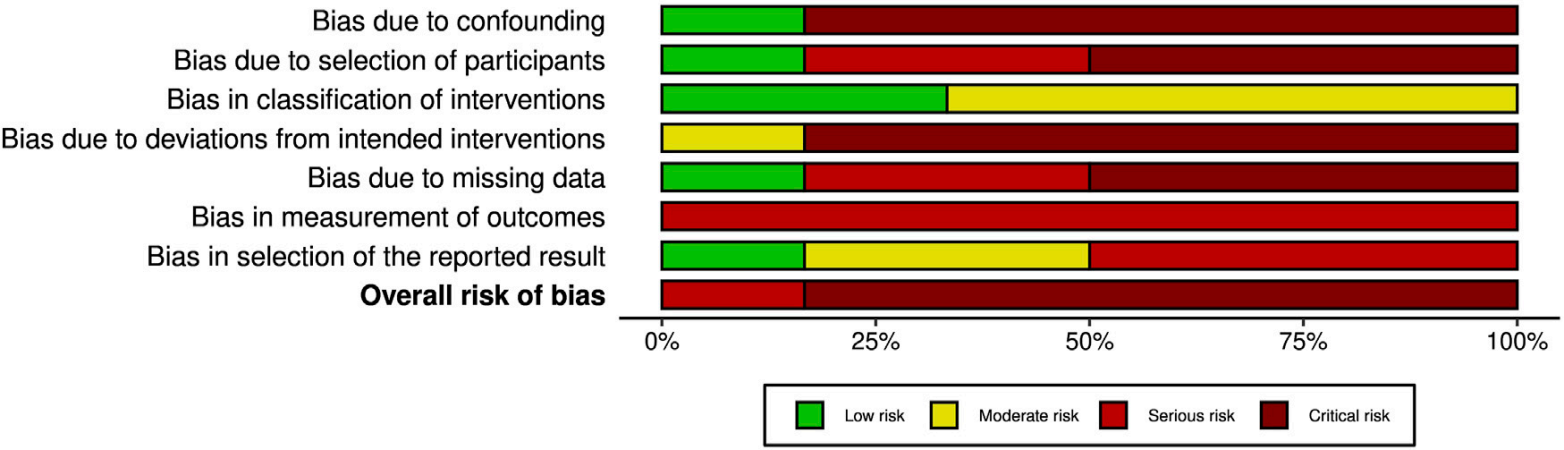
Overall risk of bias graph from assessment with the ROBINS-I tool. The graph illustrates authors’ judgements for each of the seven risk of bias categories, presented as percentage of low, moderate, serious and critical risk of bias trials.

A summary of the risk of bias assessment for included studies is shown in Figures 3–5. Risk of bias was deemed “critical” in all 5 case series [13–15, 23, 24] and “serious” in the randomised controlled trial [22], with an overall rating of “critical.” For case series, the domains associated with the highest risk of bias were confounding, selection of participants, deviations from intended interventions, and missing data. For the randomised controlled trial, most bias was attributed to lack of patient and outcome assessor blinding (bias in outcome measurement). Risk of bias justifications are provided in appendix 2 (Supplementary Material).

### Study Characteristics

Table 1 shows the characteristics of included studies. Regarding method of abdominal wall closure, two studies evaluated repair with mesh suture [22, 24] and four studies evaluated repair with mesh strip [13–15, 23]. The main indication for implantation was varied but included contaminated incisional hernia repair [14, 15], midline ventral hernia repair [15, 23, 24], laparotomy closure [15, 22, 24], and primary umbilical hernia repair [13]. Since the same branded mesh suture (Duramesh™) was used in all studies, there was no device variation [22, 24]. However, size variation was present due to differing preferences between individual surgeons. One study used solely Duramesh™ MSP300 (number 1 Prolene) [22], whereas the other study used a range of sizes, including those with filaments equivalent to 2/0, 0, 1, and 2 suture [24]. In the four mesh strip studies, all strips were cut from non-absorbable polypropylene mesh to a width of 2 cm, with variation attributable to hospital formulary (Prolene, Ethicon [13–15] or Parietene, Covidien [23]). Median follow-up of included studies was 11.9 months (range 2.7–35.3 months).

**TABLE 1:** Characteristics of included studies.

First author	Year	Country	Journal	Study type	Single surgeon vs. multiple surgeons	Study dates	Sample size	Population	Intervention	Comparator	Mean follow-up
Hackenberger [24]	2023	United States	Frontiers in Surgery	Retrospective case series	Multiple surgeons (multi-centre series)	Jan 2023 - Aug 2023	314	Abdominal wall closure (various indications)	Duramesh (various sizes used)	N/A	2.7 months
Lanier [15]	2016	United States	Plastic Reconstruction Surgery Global Open	Retrospective case series	Single surgeon	NR	107	Abdominal wall closure (various indications)	Mesh strip (Ethicon prolene)	N/A	7.7 months
Dumanian [14]	2018	United States	American Journal of Surgery	Retrospective case series	Single surgeon	Nov 2013 - Feb 2017	48	Contaminated incisional hernia	Mesh strip (Ethicon prolene)	N/A	11.8 months
Moradian [13]	2023	United States	Plastic Reconstruction Surgery Global Open	Retrospective case series	Single surgeon	Aug 2016 - Mar 2021	33	Primary umbilical hernia	Mesh strip (Ethicon prolene)	N/A	35.3 months
Berrevoet [22]	2024	Belgium	Unpublished (ongoing trial)	Randomized controlled trial	Multiple surgeons (single-centre RCT)	May 2023 - Jun 2024	65 (33 vs. 32)	Laparotomy 5 cm or greater	Duramesh (MSP300)	2/0 PDS	12 months
Sarac [23]	2024	United States	Plastic Reconstruction Surgery Global Open	Retrospective case series	Single surgeon	Sep 2013 - Dec 2022	18	Midline ventral hernia	Mesh strip (Covidien parietene)	N/A	28.3 months

Order of studies arranged by sample size. NR, not reported.

### Patient Demographics

Patient demographics are shown in Table 2. Mean age of reported patients ranged from 43 to 66 years old. Mean BMI ranged from 25 to 34 kg/m^2^. 12%–31% had diabetes, and 0%–27% were active smokers. Three studies included patients with ventral hernia widths ranging from 9.0 to 10.5 cm, which also tended to have greater operative complexity i.e., higher rates of contamination (Centers for Disease Control and Prevention (CDC) grade 2–4 [14, 15] and Ventral Hernia Working Group (VHWG) classification 3-4 [14, 15]), concurrent anterior component separation [14, 23], and/or with higher perioperative risk (American Society of Anaesthesiologists (ASA) classification 3 or above [16]). In contrast, one study investigated clean repair (CDC grade 1) of small primary umbilical hernias with defect size <3 cm [13]. Another study investigated laparotomy closure predominantly in patients with pancreatic malignancy (72.7%), who had pancreaticoduodenectomy through bilateral subcostal incision (72.7%) as the incision of choice [22]. The other study included patients having mainly ventral hernia repair or laparotomy closure (62%), but did not specify mean defect size nor laparotomy wound length [24].

**TABLE 2:** Baseline characteristics of the included population and notable study specific characteristics. Order of studies arranged by sample size.

First author	Short summary	Mean age ± SD	Male (female)	Mean BMI ± SD	Diabetes	Current smoker	Mean defect size of hernia subgroup	Study specific characteristics
Hackenberger [24]	Duramesh registry study^a^	57.3 ± 13.9	153 (226)	30.1 ± 7.0	18.5%	13.2%	NR	• 62% had ventral hernia repair or laparotomy closure • 34.1% were CDC 2, 3 or 4
Lanier [15]	Mesh strip series of abdominal wall closure	53.9 ± 14.8	38 (69)	29.0 ± 7.0	12.1%	10.3%	9.1 ± 5.5 cm (n = 41)	• 71.0% had ventral hernia repair as the index operation • 45.8% were CDC 2, 3 or 4 • 44.0% were VHWG 3 or 4 • 43% ASA 3 or above
Dumanian [14]	Mesh strip series of contaminated incisional hernia repair	62.2 ± 14.2	16 (32)	29.8 ± 7.7	31.3%	10.4%	10.5 cm (n = 48)	• 100% were CDC 2, 3 or 4 • 100% were VHWG 3 or 4 • 69% had concurrent anterior component separation
Moradian [13]	Mesh strip series of primary umbilical hernia repair	43	20 (13)	26	12.1%	9.1%	All hernia defects were ≤ 3 cm (n = 33)	• 100% were CDC 1 • Mean operating time was 69mins
Berrevoet [22]	Interim data from MOMENTUM study^b^	66.1 ± 12.0 vs. 70.5 ± 10.1	23 (10) vs. 15 (17)	25.0 ± 4.1 vs. 25.0 ± 4.9	18.1% vs. 15.6%	27.3% vs. 15.6%	N/A	• Study intended to demonstrate non-inferiority for surgical site events • 72.7% and 62.5% had malignancy, usually pancreatic • 72.7% and 71.9% had bilateral subcostal incision as the laparotomy
Sarac [23]	Mesh strip series of midline ventral hernia repair	56 ± 15	5 (13)	34 ± 14	27.8%	0%	9.0 ± 3.0 (n = 18)	• 11% were Kanters modified-VHWG 3 • 38.9% had concurrent anterior component separation

^a^ Demographic data only available for entire cohort of patients within the study, including 17.2% with indications not pertaining to abdominal wall closure.

^b^ Data presented as Duramesh™ group versus conventional suture group.

### Primary and Secondary Outcomes

Primary and secondary outcomes are reported below and raw data from each study presented in Table 3.

**TABLE 3:** Primary and secondary outcomes extracted from each study.

First author	Short summary	Incisional hernia (occurrence or recurrence)	Surgical site occurrence (SSO)	Surgical site infection (SSI)	Enterocutaneous fistula	Seroma	Haematoma	Soft tissue breakdown	Fascial dehiscence	Return to theatre	Mean length of hospital stay	Mortality
Hackenberger [24]	Duramesh registry study	0.6%	17.9%	6.1%	0.3%	4.5%	1.0%	3.5%	1.6%	5.7%	5.3 days	NR
Lanier [15]	Mesh strip series of abdominal wall closure	3.7%	16.8%	4.6%	0%	10.3%	3.7%	0%	NR	5.6% at 1 month	NR	1.9%
Dumanian [14]	Mesh strip series of contaminated incisional hernia repair	6%	27%	19%	0%	NR	2.1%	NR	NR	10.4% at 1 month	NR	2.1%
Moradian [13]	Mesh strip series of primary umbilical hernia repair	3.0%	3.0%	3.0%	0%	0%	0%	0%	0%	0% at 1 month	NR	NR
Berrevoet [22]	Interim data from MOMENTUM study	0% vs. 0%	0% vs. 0%	0% vs. 0%	0% vs. 0%	0% vs. 0%	0% vs. 0%	0% vs. 0%	0% vs. 0%	3.0% vs. 3.1% at 12 months	15.2 ± 7.1 vs. 15.2 ± 6.1 days	3.0% vs. 3.1%
Sarac [23]	Mesh strip series of midline ventral hernia repair	50%	50%	16.7%	0%	11.1%	0%	33.3%	0%	11.1%	3.4 ± 1.5 days	NR

Order of studies arranged by sample size. NR, not reported.

### Incisional Hernia

**FIGURE 6:**
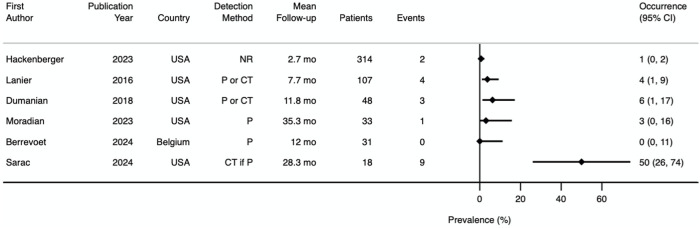
Prevalence Forest plot demonstrating incisional hernia occurrence. Studies are ordered by sample size. NR, not reported; P, physical examination; P or CT, physical examination or CT scan; CT if P, CT scan if physical examination positive; mo, months.

Our pre-specified primary outcome was incisional hernia at 1 year and 3 years. Only one study prospectively reported incisional hernia occurrence at 1 year [22] and no study prospectively reported incisional hernia occurrence at 3 years. All other studies reported incisional hernia occurrence retrospectively, with mean follow-up ranging from 2.7 to 35.3 months [13–15, 23, 24]. A prevalence Forest plot with 95% confidence intervals (CI) is shown in Figure 6. The method for incisional hernia detection was clinical diagnosis in two studies [13, 22], clinical or CT scan diagnosis in two studies [14, 15], CT scan diagnosis if physical examination was positive in one study [23], and unspecified in one study [24]. The median incisional hernia occurrence rate was 3.4% (range 0%–50%). Subgroup analysis of incisional hernia rate after either ventral hernia repair or laparotomy closure revealed 16 incisional hernias (9.8%) after 163 ventral hernia repairs (excluding simple umbilical) [14, 23, 24] and 1 incisional hernia (0.8%) out of 126 patients who had laparotomy closure.

### Surgical Site Occurrence

**FIGURE 7:**
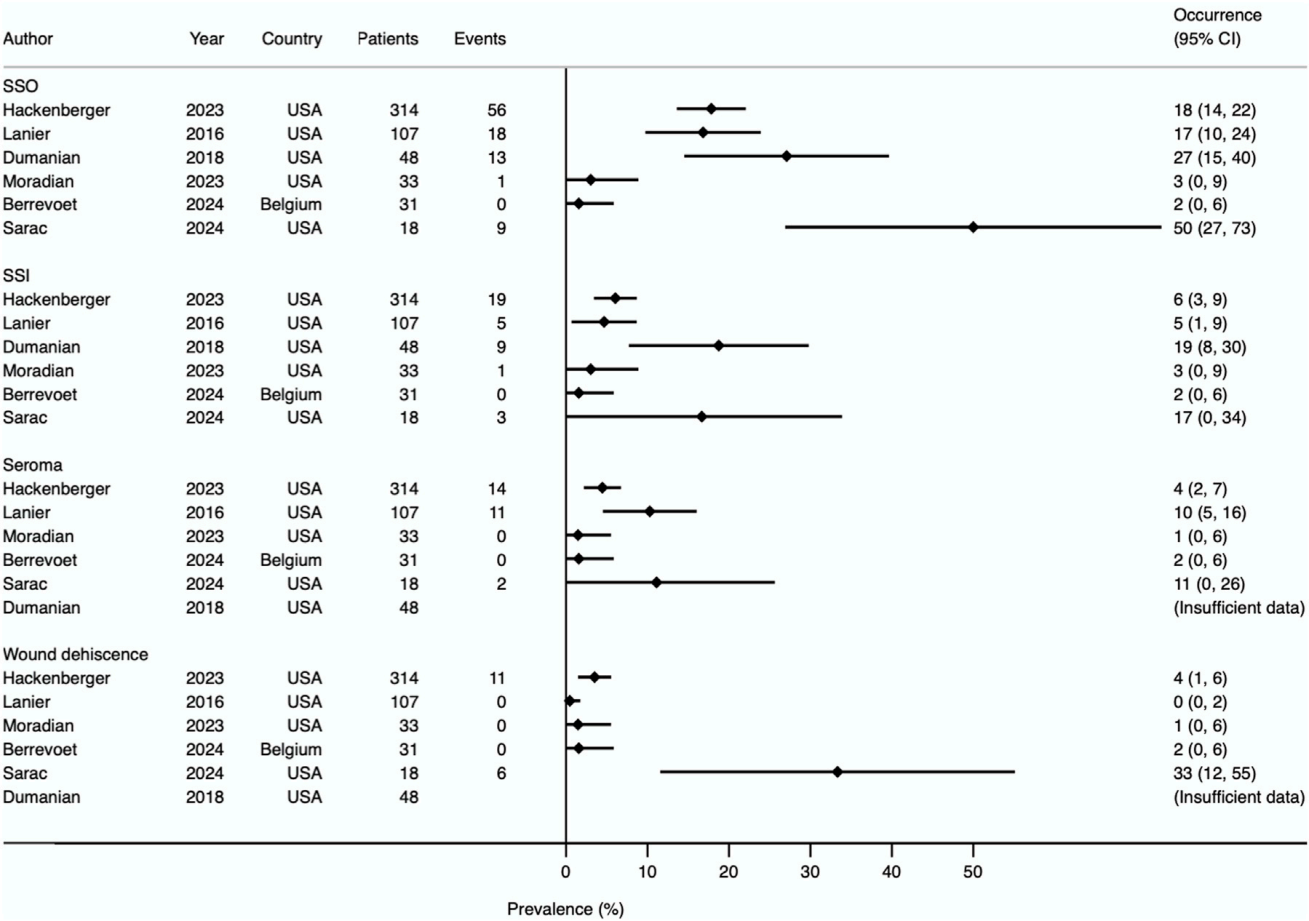
Prevalence Forest plot demonstrating occurrence rates of various secondary outcomes. Studies are ordered by sample size. SSO, surgical site occurrence; SSI, surgical site infection.

**FIGURE 8:**
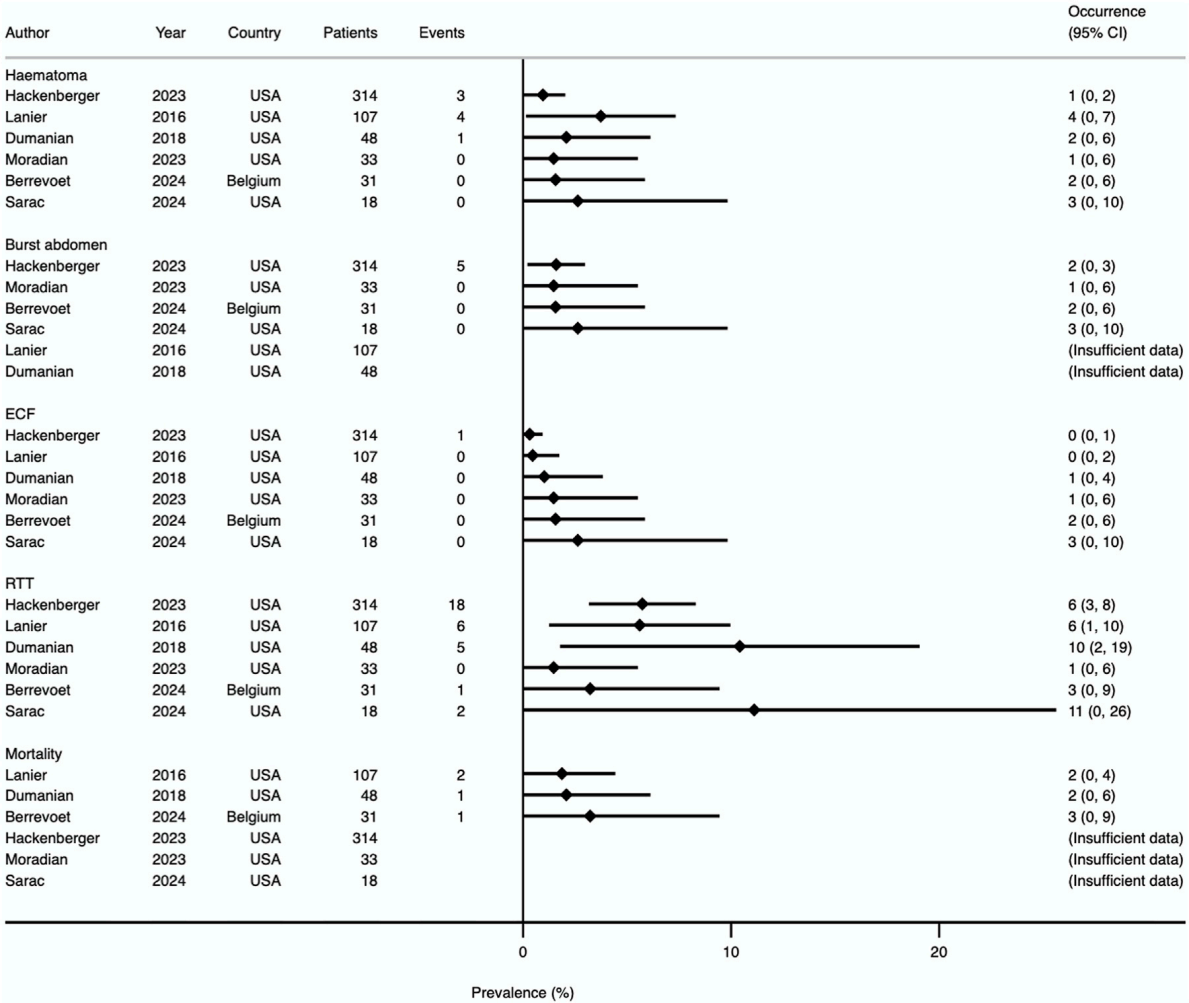
Prevalence Forest plot demonstrating occurrence rates of various secondary outcomes. Studies are ordered by sample size. ECF, enterocutaneous fistula; RTT, return to theatre.

Five studies reported surgical site occurrence (SSO) [13–15, 22, 23]. One study provided data for surgical site events and surgical site infection (SSI) separately, which were summed to obtain the value for SSO [24]. Therefore, SSO rates could be obtained from all 6 studies. Prevalence Forest plots with 95% CI are shown in Figures 7, 8. Pathologies included within the SSO umbrella term were consistent between all studies, but there was variation in how timing was defined. Two studies defined SSO as occurring within 3 months [23, 24], one study using 1 month [22], and three studies did not specify [13–15]. The median rate of SSO was 17% (range 0%–50%). When investigating ventral hernia repair (excluding simple umbilical) and laparotomy closure subgroups, the SSO rate was 31% [14, 23, 24] and 8.7% [22, 24] respectively. Median occurrence rates from individual secondary outcomes are shown in Table 4.

**TABLE 4:** Secondary outcomes with corresponding median value and number of studies reporting each outcome.

Secondary outcome	Median occurrence rate (range)	Number of studies
Surgical site occurrence (SSO)	17.4% (0%–50%)	6
Surgical site infection (SSI)	5.4% (0%–19%)	6
Seroma	4.5% (0%–11.1%)	5
Haematoma	0.5% (0%–3.7%)	6
Soft tissue breakdown	0% (0%–33.3%)	5
Burst abdomen	0% (0%–1.6%)	4
Enterocutaneous fistula	0% (0%–0.3%)	6
Mortality	2.1% (1.9%–3.0%)	3

### Return to Theatre

All 6 studies reported theatre returns (results shown in Figure 8). Three studies defined this as occurring within 30 days [13–15], one within 12 months [22], and two did not specify [23, 24]. In total, 33 of 553 patients (6.0%) returned to theatre within the timeframe defined by each study. Four studies allowed for further sub-group analysis, relating to wound and intraabdominal complication rates [14, 22–24]. Data were available for 27 patients, of which 15 (56%) had a reoperation for a wound complication and 12 (44%) for an intraabdominal-related event.

### Length of Hospital Stay

Length of hospital stay was reported by three studies [22–24]. Mean length ranged from 3.4 to 15.2 days.

### Mortality

Mortality was reported by three studies [14, 15, 22]. There were 4 deaths from 188 patients (2.1%). Mortality is included in the Forest plot in Figure 8.

### Patient Reported Outcome Measures

Patient reported outcome measures (PROMs) were reported by 2 studies [13, 22]. One study conducted a telephone questionnaire, achieving a response rate of 61% (20/33) [13]. Of these 20 respondents, 18 were unable to feel a knot, 15 were satisfied with cosmesis and 16 reported improved quality of life. Validated quality of life questionnaires were not used and options to questions were either “yes” or “no.” In addition, pain scores at 7 days were assessed by a 11-point numeric rating scale. Eighteen of 20 had pain score of 0, 1 patient had pain score of 10, and 1 did not provide a response.

The other study compared patient satisfaction and pain score at 1 month between intervention and control arms, using a five-point “smiley face” scale and 11-point numeric rating scale respectively [22]. For this outcome, per protocol analysis was conducted on 62 of 65 patients (31 in each arm) due to 1 mortality and 2 reoperations with a different reclosure method to the original randomised group. Patient satisfaction (mean ± SD; 4.9 ± 0.4 vs. 4.9 ± 0.3, p = 0.72) and pain score at rest (mean ± SD; 0.8 ± 1.4 vs. 0.6 ± 0.8, p = 0.62) showed no statistically significant difference between the Duramesh^™^ and conventional suture groups.

## Discussion

The most important long-term surgical complication after failed closure of midline laparotomy or ventral hernia repair is occurrence or recurrence of incisional hernia, which can reduce quality of life significantly. Our systematic review assessed incisional hernia occurrence after repair with the novel mesh suture, Duramesh™, or its mesh strip precursor.

The mechanism behind mesh suture/strips is thought to be attributable to physics and the concept of suture pull-through. By definition, pressure equals force divided by surface area, so when tissue edges are approximated, local pressure at the suture-tissue interface is determined by outward force (rectus contraction and intraabdominal pressure) and the surface area of contact [25]. Mesh suture/strips are thought to reduce the likelihood of chronic “cheese-wiring” due to broader surface area of contact and enhanced tension distribution, therefore resisting fascial dehiscence and development of subsequent incisional hernia [9]. In an experimental study, Cengiz et al. demonstrated that smaller tissue bites increased wound strength by lowering the tension on individual stitches [26]. The principle underlying Duramesh™ is similar to the “small-bite closure” method and mesh repair of incisional hernia, as these techniques aim to distribute tension evenly along closed fascial edges. Furthermore, the 3D macroporous structure of Duramesh™, akin to other permanent mesh prostheses, has demonstrated greater tissue integration in *in-vivo* studies, suggesting an additional tissue response around a mesh scaffold via a permanent foreign body scar response [11, 27].

We found considerable study heterogeneity, poor study quality and critical risk of bias in this systematic review. Direct comparison of incisional hernia occurrence with selected articles from available high-quality literature was not possible due to heterogeneity in reporting follow-up, with a range of short to medium-term follow-up observed. However, incisional hernia occurrence with mesh suture/strips did appear lower than known published estimates. The STITCH trial revealed an incisional hernia rate of 13% at 1 year (n = 268, 9%–18% 95% CI) for the “small bite” group after midline laparotomy [28], and rates were 13% and 18% at 2 years (n = 188, 8%–18% 95% CI; n = 185, 13%–24% 95% CI) in the prophylactic onlay and sublay mesh groups of the PRIMA trial, respectively [6]. Other historic figures include a 21% recurrence rate at 21 months median follow-up (n = 3258) from the Danish registry study [29], and 18% at 1 year (n = 84, 10%–28% 95% CI) after primary mesh repair from Luijendijk et al’s landmark trial of mesh versus suture repair of incisional hernia [30]. The patient cohorts from these studies had largely similar characteristics (mean age, mean BMI, diabetics, smokers) to those found in this systematic review, excepting patients in the PRIMA trial who underwent elective abdominal aortic aneurysm repair and were thus increasingly prone to incisional hernia development due to associated connective tissue disease.

The STITCH [28] and PRIMA trials [6] were sufficiently important to achieve Lancet publication and have impacted the way surgeons perform closure of the abdominal wall following primary laparotomy. Notably, both studies involved other surgical specialties including gynaecology, urology and vascular surgery. Rates of incisional hernia amongst participating institutions in the STITCH trial [28] were varied (0%–25%) possibly reflecting differences in abdominal closure expertise and patient case mix rather than suture technique alone. Learning curve was also difficult to measure and may not have been accounted for. Conversely, in this systematic review of mesh suture/strips, general surgeons with an abdominal wall interest were performing closure which may explain the low incisional hernia rates observed compared to STITCH. Another common criticism of STITCH [28] is whether the use of a 31 mm needle (2/0 PDS) in the small bite arm versus a 48 mm needle (1 loop PDS) in the large bite arm contributed to the observed differences due to greater “buttonholing” of fascia with larger sized needles. The studies in this systematic review also used a range of needle sizes (from 2/0–2) so its specific impact remains uncertain. However, pre-clinical studies have shown that Duramesh™ has a reduced tendency to cut through tissue once implanted [10]. Applying only light suture tension to approximate tissues and avoid strangulation is a principle discussed in contemporary guidelines [7] and should be adhered to no matter the product used.

Prophylactic mesh augmentation during the index operation has garnered attention but despite the evidence has not been widely adopted due to impracticality. The PRIMA trial [6] was performed in patients with abdominal aortic aneurysm repair without violation of the gastrointestinal tract. Theoretical risk of contamination is often anecdotally cited as a reason for not performing prophylactic mesh augmentation and in two of the case series identified in this systematic review [14, 15], this concern was one of the indications for using mesh suture/strips instead. The ideal method of abdominal wall closure should prevent incisional hernia while minimising SSO risk and additional implantation time, particularly in comorbid patients where shorter operative times are beneficial. This is a potential strength of Duramesh™, but its incisional hernia and SSO rates need to be validated further with high-quality and unbiased prospective studies. The PRIMA trial [6] found only onlay prophylactic mesh to be statistically superior to primary suture. Onlay mesh placement, despite being relatively easy to implant, is considered an inferior biomechanical repair compared to retromuscular mesh placement for ventral and incisional hernias [8]. This perceived inferiority may have limited its widespread adoption for laparotomy closure. Duramesh™ may offer a pragmatic alternative particularly with patient populations where minimising operative complexity is crucial.

An important confounding factor in this review is length of follow-up. Nearly half of incisional hernias in a study by Fink et al. occurred more than a year after index operation, with rates rising from 12.6% at 1 year to 22.4% at 3 years post-surgery [31]. Burger et al. showed that recurrence following hernia repair continued even up to 10 years after surgery, particularly in suture repaired cases where the 10-year cumulative rate of recurrence was 63% for suture repair and 32% for prosthetic repair [32]. The follow-up duration of studies included in our systematic review ranged from 2.7–35.5 months, with four studies having 1 year follow-up or less [14, 15, 22, 24]. It is possible that shorter follow-up contributed to the relatively low incisional hernia occurrence. The series by Sarac [23] and Moradian [13], which had greater than 2 years mean follow-up, showed conflicting results with recurrence rates of 50% and 3% respectively. However, it should be noted that the patients studied were fundamentally different, comprising large midline ventral hernias in Sarac’s series [23] compared with small umbilical hernias in Moradian’s series [13]. It can therefore be argued that differences in operative complexity, BMI, CDC and VHWG grade meant that these patients were more likely to develop incisional hernia. Of note, the mesh strips in Sarac’s cohort were inserted using a Pulvertaft tendon weaver [23] rather than the “guiding suture”, as described originally by Dumanian [33]. Whether this has a significant impact compared to the differences in risk factors is unknown. Smoothness of mesh strip passage and tissue trauma is difficult to quantify and were not reported in any of the studies. We emphasise a need for continued long-term follow-up for patients treated with mesh suture/strips.

Variation in definitions and detection of incisional hernia may be another confounding factor in our review. A universally accepted definition for recurrence detection remains elusive, but recently a meeting of key opinion-leading hernia surgeons used a nominal group technique, which revealed that CT scanning should be the diagnostic method of choice [34]. Ethical issues around ionising radiation are greatly reduced by modern scanners but routine uptake in hernia trials remains a challenge [35]. An additional issue is whether small (<1 cm) fascial discontinuities visible on CT but without any definite intraabdominal protrusion should be classified as recurrence. Nonetheless, it is well-established that imaging increases the proportion of incisional hernia since impalpable recurrence is identified [36]. Although it may be argued that asymptomatic hernias are clinically irrelevant, Bloemen et al. propose that reporting imaging-detected hernias is justified since they are most at risk of incarceration, and some may enlarge over time or become symptomatic [37]. Notably, the STITCH trial, protocolled routine ultrasonography follow-up, with review of additional CT scanning if performed on clinical grounds [28]. In contrast, our review found imaging was not employed routinely and was only selectively used in three studies following equivocal physical examination. This may partly explain lower incisional hernia observed and emphasises the need for precise diagnosis for hernia trials.

The potential for larger volume knots when using mesh suture/strips compared to conventional sutures, raises concerns regarding patient comfort and SSO risk. The MOMENTUM trial [22], our only head-to-head comparative study between Duramesh™ and small-bite PDS closure, has so far demonstrated non-inferiority of Duramesh™ for SSO, pain score and patient satisfaction, potentially suggesting it is safe in the short-term (1 year follow-up). Marangi et al’s randomised controlled trial (RCT) of rectus diastasis repair [38], although excluded from our review, found Duramesh™ non-inferior to 0 Prolene for SSOs (including diastasis recurrence), pain score, and cosmetic satisfaction, using the validated BODY-Q score [39], but again follow-up duration was short at 6 months. Most patients in Moradian’s series of primary umbilical hernias were unable to feel the knot [13], although this aspect warrants further investigation in high-quality interventional trials. Other studies have shown that palpable sutures and stitch granulomas can occur with retained suture material and a thin subcutaneous fat layer around the umbilicus [40]. The median SSO result (17.4%) from our systematic review suggests mesh suture/strips may be similar to SSO rates reported in high-quality randomised controlled trials of prophylactic mesh repair of laparotomy, small-bite closure, and mesh repair of incisional hernia [6, 25, 27]. However, as can be seen in Figure 7, confidence intervals are wide with considerable heterogeneity between studies, preventing meta-analysis. As follows, there is currently insufficient high-quality and unbiased data to form a firm conclusion regarding its safety. Moreover, questions remain regarding the superiority of mesh suture over planar mesh for SSI rates. There are clear differences such as surface area of implant, pore size, tissue trauma, and devascularisation of tissue planes, which may theoretically lead to differences in SSI prevalence. Albeit substantially less invasive than planar mesh repair, the possibility of chronic mesh suture infection leading to explantation remains a critical area for future research.

An inherent limitation of our systematic review is that included trials were retrospective single-arm uncontrolled studies with critical risk of bias. Thus, patients were not randomised, and causality cannot be established. These were the best available data but multiple biases including selection bias and missing data bias are likely present. In the primary analysis, all data were pooled to compute a median value, and no distinction was made between patient variables such as hernia defect size, VHWG grade and ASA grade. Also, surgical variables such as surgeon experience, needle size and surgical technique may confound outcomes, and we suggest they should be standardised for future studies. Moreover, we were unable to adjust for patient risk factors, such as age, body mass index, or chronic obstructive pulmonary disease. Despite these limitations, the strength of this review is the use of a systematic approach and assessment of study quality using risk of bias tools that require rigorous methodology.

In summary, our systematic review found that mesh suture and mesh strips may be associated with low rates of short to medium-term incisional hernia occurrence, but this is merely anecdotal at this stage. Consequently, due to poor data quality, there is currently insufficient evidence to make any recommendations regarding Duramesh™ over current strategies for abdominal wall closure. Similarly, not enough high-quality data exists regarding product safety, so its safety profile remains uncertain. Well-designed RCTs with long-term follow-up are required to generate strong evidence. The abdominal wall community eagerly awaits the full results of the MOMENTUM study [22] and currently other RCTs are also recruiting [41, 42]. We suggest that future RCTs adhere to minimum standards [34] including at least 12 months of follow up, uniform technique for Duramesh™ implantation, and consistent use of imaging for hernia detection. High-quality comparative studies evaluating cost-effectiveness, quality of life, and other patient reported outcome measures are also required. Until then, Duramesh™ remains a potentially useful tool, but should be considered as part of a broader strategy of hernia prevention tailored to individual patient risk profiles and surgical contexts.
