# Supplementary material for: Mesh Suture and Mesh Strips to Prevent Incisional Hernia Following Abdominal Wall Closure or Ventral Hernia Repair: Systematic Review
Source: J Abdom Wall Surg. 2025 May 14;4:14573. doi: 10.3389/jaws.2025.14573 (PMC12120353; doi:10.3389/jaws.2025.14573)
Supplement: Supplementary file 1 [file DataSheet2.DOCX]

## Appendix 2

**Authors’ justifications for risk of bias conclusions**

**Introduction**

Risk of bias of included case series were assessed using an adapted form of the ROBINS-I tool. Risk of bias assessment for the MOMENTUM study was performed using both the ROBINS-I tool and the Cochrane RoB2 tool. We felt that a consistent assessment with the same risk of bias tool would be more meaningful as it enables between-study comparison for all studies included in this systematic review. The Cochrane RoB2 tool was also used since it is the gold standard bias assessment tool for randomised controlled trials. It is similar to the ROBINS-I tool other than the randomisation domain which we comment on later.

**Commentary on risk of bias assessment with ROBINS-I**

The ROBINS-I tool allows for assessment of non-randomised studies where there are two or more intervention groups. Since case series contain only one intervention arm, we adapted the ROBINS-I tool and hypothesized situations whereby similar case series for standard treatment (e.g. suture closure of laparotomy or mesh repair of ventral hernia) were conducted simultaneously and in parallel to the interventional case series, with the same characteristics as individual mesh suture/strip studies. For example, for Moradian’s case series of mesh strip repair for small umbilical hernia, a control arm (suture repair of umbilical hernia) with the same recruitment characteristics was hypothesised. Therefore, consecutive patients selected and operated on by a single surgeon would be inputted onto a database and outcome data retrospectively extracted/analysed at the same timepoint as patients in the mesh strip arm. This hypothetical retrospective cohort study, with an interventional and control arm, was then compared to the “target trial” in the usual manner for ROBINS-I. Our “target trial” was a randomised controlled trial of Duramesh vs. conventional treatment for closure of laparotomy or ventral hernia repair with at least 1000 patients recruited with a variety of different indications for generalisability. The outcome of interest was incisional hernia. Below are the risk of bias justifications:

| **Risk of bias domain** | **Comments** |
| --- | --- |
| Domain 1: Bias due to confounding | Uncontrolled confounding inherent in retrospective studies. Recruitment of patients highly selected and no adjustments were made for confounders which could distort study outcomes. Confounders include BMI, smoking, diabetes, hernia defect size, comorbidities, and socioeconomic status. All case series were graded as critical risk of bias. Well-conducted RCTs have low risk of bias from confounding due to randomization of patients. MOMENTUM study had even distribution of patients with similar BMI, diabetics, smokers and other comorbidities between groups (p>0.05). |
| Domain 2: Bias due to selection of participants | Selection into case series likely to be influenced by knowledge of cofounders and outcome. Those retrospective series that specified any exclusion criteria meant that, by definition, selection of patients occurred and were hence graded as critical risk of bias. Well-conducted RCTs have low risk of this bias due to its prospective nature, randomization and allocation concealment mechanisms. |
| Domain 3: Bias in classification of interventions | There is little ambiguity between what constitutes mesh suture, mesh strip and conventional suture. Hackenberger’s study identified patients through institutional implant logs of Duramesh with publication of raw data, so unlikely to be susceptible to differential or non-differential misclassification. Other case series used prospectively maintained databases but did not specify any further so cannot be considered low risk of bias. It is not known how/when patients were inputted onto the database, for instance, whether performed at the time of operation or retrospectively based on discharge papers or clinical codes. Well-conducted RCTs have low risk of bias from classification issues as intervention groups are actively assigned. |
| Domain 4: Bias due to deviations from intended interventions | All retrospective studies are inherently susceptible to this type of bias and hence were graded as critical. Patients were not prospectively allocated to intervention groups, and therefore the operating surgeon can at any time pre-operatively and/or intra-operatively, decide whether to use mesh suture/strip or other technique to close the abdominal wall based on judgement. MOMENTUM RCT was unblinded and randomisation occurred in the operating room before knife to skin. Little further information is included, so whilst unlikely to cause bias, cannot be graded as low risk. |
| Domain 5: Bias due to missing data | Missing data for retrospective studies highly susceptible to missing data bias. Outcomes were retrospective chart reviews and not collected at pre-specified time-points during the follow-up period for the purposes of a trial. No per-protocol data collection form used, so some clinicians may only report clinical outcomes they believe to be relevant rather than according to strict definitions. Also, follow-up duration not equal between patients therefore, by definition, under-reporting present. Studies with >2 years mean follow-up slightly less likely to under-report incisional hernia but still high risk. MOMENTUM trial low risk of missing data bias as patients were followed up at pre-defined timepoints and eCRF used for data collection. |
| Domain 6: Bias in measurement of outcomes | All outcome assessors in retrospective studies would be aware of the intervention received i.e. mesh suture or hypothetical standard treatment. Therefore, highly susceptible to bias in detection and diagnosis of incisional hernia for equivocal cases. Also, outcome assessors may be clinicians not involved in the study and have differing understanding of reporting definitions. However, in each case series secondary outcome definitions were well defined. Patients and outcome assessors in MOMENTUM study were unblinded so detection bias likely. |
| Domain 7: Bias in selection of reported result | Risk of bias for the retrospective studies where incisional hernia was an outcome are less likely to selectively report, since it is the most important surgical outcome in abdominal wall surgery and should always be reported. However, these studies cannot be graded as low risk of bias as there is no clear evidence of a registered protocol or *a priori* suggestion of method for data analysis. Studies where reporting of incisional hernia can be either physical examination or CT are susceptible to reporting bias. MOMENTUM study had a registered protocol on clinicaltrials.gov. |

**Commentary on the randomisation risk of bias analysis for MOMENTUM**

We used the Cochrane RoB2 tool to analyse the MOMENTUM trial as it is the gold standard bias assessment tool for randomised controlled trials. The only significant difference between this tool and the ROBINS-I tool is the randomisation domain. The ROBINS-I tool replaces the randomisation domain with 2 other domains, namely, bias due to confounding and bias due to selection of participants. These are generally graded as low risk for well-performed randomised controlled trials. MOMENTUM used 3:3 block randomisation using an electronic random number generator. A third-party (Archer Research) was used for allocation concealment and subsequent trial management. Baseline differences between groups did not suggest a problem with the randomisation process. Therefore, it was graded as low risk of bias for randomisation.
