# Supplementary material for: Mesh Suture and Mesh Strips to Prevent Incisional Hernia Following Abdominal Wall Closure or Ventral Hernia Repair: Systematic Review
Source: J Abdom Wall Surg. 2025 May 14;4:14573. doi: 10.3389/jaws.2025.14573 (PMC12120353; doi:10.3389/jaws.2025.14573)
Supplement: Supplementary file 2 [file DataSheet1.DOCX]

## Appendix 1

Our search strategy for each electronic database is provided below.

**Database: Ovid MEDLINE <2000 to February 28, 2024>**
**Search Strategy:**
**1**  Suturable mesh.ti,ab,kf,kw. (3)
**2**  Mesh suture*or mesh-suture*.ti,ab,kf,kw. (0)
**3**  Mesh suture*.ti,ab,kf,kw. (83)
**4**  Mesh strip*.ti,ab,kf,kw. (27)
**5**  (Duramesh or Dura-Mesh or Dura Mesh or Duomed).ti,ab,kf,kw. (5)
**6**  1 or 3 or 4 or 5 (113)
**7**  (Laparotom* or Minilaparotom* or abdominal surgery).ti,ab,kf,kw. (73965)
**8**  exp Laparotomy/ (20437)
**9**  7 or 8 (82928)
**10**  (Digestive System Surgical Procedure* or Gastrointestinal Surgical Procedure*).ti,ab,kf,kw. (154)
**11**  exp Digestive System Surgical Procedures/ (425322)
**12**  10 or 11 (425397)
**13**  (Abdominal wall reconstruction or Abdominal Closure Technique* or Abdominal wound closure technique*).ti,ab,kf,kw. (1332)
**14**  exp Herniorrhaphy/ or exp Hernia, Ventral/ (20529)
**15**  exp Abdominal Wound Closure Techniques/ (913)
**16**  exp Abdominal Wall/ (7816)
**17**  exp Hernia, Abdominal/ (30776)
**18**  13 or 14 or 15 or 16 or 17 (42689)
**19**  Ventral hernia*.ti,ab,kf,kw. (4065)
**20**  exp Incisional Hernia/ (1593)
**21**  Incisional hernia*.ti,ab,kf,kw. (5411)
**22**  Postoperative Hernia*.ti,ab,kf,kw. (312)
**23**  (Umbilical hernia repair* or Umbilical Hernia Repair Surgery).ti,ab,kf,kw. (406)
**24**  exp Hernia, Umbilical/ (4097)
**25**  23 or 24 (4248)
**26**  20 or 21 (5810)
**27**  19 or 22 or 25 or 26 (13203)
**28**  18 or 27 (45910)
**29**  9 or 12 (494815)
**30**  28 or 29 (533126)
**31**  6 and 30 (63)
**32**  exp Animals/ (27082843)
**33**  exp Humans/ (21874177)
**34**  32 not 33 (5208666)
**35**  31 not 34 (52)
**36**  (new or novel).ti,ab,kf,kw. (4547107)
**37**  31 and 36 (22)
**38**  37 not 34 (17)

**Database: CENTRAL <2000 to 2024 February 28>**
**Search Strategy:**

| Search number | Query | Results |
| --- | --- | --- |
| 33 | #32 not #29 | 308 |
| 32 | #26 and #31 | 639 |
| 31 | (new or novel) | 5,883,599 |
| 30 | #26 not #29 | 306 |
| 29 | #27 or #28 | 27,193,678 |
| 28 | "humans"[All Fields] | 22,018,233 |
| 27 | "animals"[All Fields] | 7,541,226 |
| 26 | #5 and #25 | 2,998 |
| 25 | #23 or #24 | 2,562,637 |
| 24 | #6 or #7 | 332,324 |
| 23 | #14 or #22 | 2,287,900 |
| 22 | #15 or #19 or #20 or ##21 | 2,257,433 |
| 21 | #16 or #18 | 6,415 |
| 20 | ((Umbilical hernia repair* or Umbilical Hernia Repair Surgery)) OR ("umbilical hernia repair"[All Fields]) | 1,662 |
| 19 | Postoperative Hernia* | 26,969 |
| 18 | Incisional hernia* | 6,415 |
| 16 | "incisional hernia"[All Fields] | 4,951 |
| 15 | Ventral hernia* | 10,036 |
| 14 | #8 or #9 or #10 or #11 or #12 or #13 | 48,549 |
| 13 | "abdominal hernia"[All Fields] | 3,183 |
| 12 | "abdominal wall"[All Fields] | 26,317 |
| 11 | "abdominal wound closure techniques"[All Fields] | 896 |
| 10 | "ventral hernia"[All Fields] | 8,888 |
| 9 | "herniorrhaphy"[All Fields] | 13,727 |
| 8 | (Abdominal wall reconstruction or Abdominal Closure Technique*) | 9,798 |
| 7 | ((Digestive System Surgical Procedure* or Gastrointestinal Surgical Procedure*)) OR ("digestive system surgical procedures"[All Fields]) | 93,867 |
| 6 | ((Laparotom* or Minilaparotom* or abdominal surgery)) OR ("laparotomy"[All Fields]) | 251,681 |
| 5 | #1 or #2 or #3 or #4 | 4,640 |
| 4 | (Duramesh or Dura-Mesh or Dura Mesh or Duomed) | 216 |
| 3 | Mesh strip* | 297 |
| 2 | Mesh suture*or mesh-suture* | 3 |
| 1 | Suturable mesh | 4,233 |

**Database: Embase <2000 to 2024 February 28>**
**Search Strategy:**
**1**  Suturable mesh.ti,ab,kf,kw. (9)
**2**  Mesh suture*or mesh-suture*.ti,ab,kf,kw. (0)
**3**  Mesh strip*.ti,ab,kf,kw. (43)
**4**  (Duramesh or Dura-Mesh or Dura Mesh or Duomed).ti,ab,kf,kw. (14)
**5**  1 or 2 or 3 or 4 (62)
**6**  (Laparotom* or Minilaparotom* or abdominal surgery).ti,ab,kf,kw. (105572)
**7**  exp laparotomy/ (100105)
**8**  (Digestive System Surgical Procedure* or Gastrointestinal Surgical Procedure*).ti,ab,kf,kw. (296)
**9**  exp gastrointestinal surgery/ (434448)
**10**  exp abdominal surgery/ (927700)
**11**  6 or 7 (151219)
**12**  8 or 9 or 10 (927769)
**13**  (Abdominal wall reconstruction or Abdominal Closure Technique*).ti,ab,kf,kw. (1832)
**14**  exp abdominal wall defect/ or exp abdominal wall/ or exp abdominal wall hernia/ (90240)
**15**  13 or 14 (90479)
**16**  Abdominal wound closure technique*.ti,ab,kf,kw. (41)
**17**  Ventral hernia*.ti,ab,kf,kw. (6052)
**18**  exp abdominal wall hernia/ (42298)
**19**  exp incisional hernia/ (9641)
**20**  17 or 18 or 19 (50072)
**21**  Incisional hernia*.ti,ab,kf,kw. (8701)
**22**  Postoperative Hernia*.ti,ab,kf,kw. (399)
**23**  21 or 22 (9041)
**24**  11 or 12 or 15 or 16 or 20 or 23 (1004046)
**25**  5 and 24 (37)
**26**  exp animal/ (31540817)
**27**  exp human/ (26313607)
**28**  26 not 27 (5227210)
**29**  25 not 28 (29)

**Cochrane Database**

Date Run: 28/02/2024 10:34:45

ID Search Hits

#1 Suturable mesh 2

#2 Mesh suture*or mesh-suture* 0

#3 Mesh suture* 1040

#4 Mesh strip* 235

#5 (Duramesh or Dura-Mesh or Dura Mesh or Duomed) 53

#6 #1 or #2 or #3 or #4 or #5 1274

#7 (Laparotom* or Minilaparotom* or abdominal surgery) 25665

#8 MeSH descriptor: [Laparotomy] explode all trees 1011

#9 #7 or #8 25665

#10 (Digestive System Surgical Procedure* or Gastrointestinal Surgical Procedure*) 4360

#11 MeSH descriptor: [Digestive System Surgical Procedures] explode all trees 20318

#12 #10 or #11 23055

#13 (Abdominal wall reconstruction or Abdominal Closure Technique*) 1050

#14 MeSH descriptor: [Herniorrhaphy] explode all trees 785

#15 MeSH descriptor: [Hernia, Ventral] explode all trees 494

#16 MeSH descriptor: [Abdominal Wound Closure Techniques] explode all trees 141

#17 MeSH descriptor: [Abdominal Wall] explode all trees 215

#18 MeSH descriptor: [Hernia, Abdominal] explode all trees 2351

#19 #13 or #14 or #15 or #16 or #17 or #18 3575

#20 Ventral hernia 733

#21 MeSH descriptor: [Incisional Hernia] explode all trees 288

#22 Incisional hernia* 1189

#23 Postoperative Hernia* 5109

#24 (Umbilical hernia repair* or Umbilical Hernia Repair Surgery) 239

#25 MeSH descriptor: [Hernia, Umbilical] explode all trees 85

#26 #24 or #25 261

#27 #21 or #22 1189

#28 #20 or #23 or #26 or #27 5756

#29 #19 or #28 7061

#30 #9 or #12 45506

#31 #29 or #30 49823

#32 #6 and #31 742

#33 animals 28999

#34 humans 896735

#35 #33 not #34 5303

#36 #32 not #35 740

#37 (new or novel) 257915

#38 #32 and #37 252

#39 #38 not #35 250

**WHO International Clinical Trials Registry Platform**

Date Performed: 28/02/2024

Mesh suture OR Duramesh (109)

**ClinicalTrials.gov Platform**

Date Performed: 28/02/2024

Mesh suture OR Duramesh (63)
